# Perioperative Outcomes With Direct Oral Anticoagulants in Orthopaedic Trauma Surgery: A Systematic Review and Meta-Analysis

**DOI:** 10.7759/cureus.97688

**Published:** 2025-11-24

**Authors:** Olive Kyaw, Chan Khin

**Affiliations:** 1 Trauma and Orthopaedics, University Hospitals Sussex NHS Foundation Trust, Brighton, GBR; 2 Trauma and Orthopaedics, St George’s University Hospitals NHS Foundation Trust, Brighton, GBR

**Keywords:** bleeding complications, direct oral anticoagulant, hip fracture, orthopaedic trauma, perioperative management, surgical delay, systematic review

## Abstract

The perioperative management of direct oral anticoagulants (DOACs) in orthopaedic trauma is variably practised. We conducted a Preferred Reporting Items for Systematic Reviews and Meta-Analyses-concordant systematic review and meta-analysis of adult trauma surgery comparing DOAC exposure with vitamin K antagonists or no anticoagulation. MEDLINE, Embase, and CENTRAL were searched (2010-October 2025) for randomised and observational studies. Pre-injury DOAC use informed analyses of mortality, transfusion, and operative delay, whereas postoperative prophylaxis trials addressed venous thromboembolism (VTE); these represent two distinct clinical questions that were analysed separately. Risk of bias (Risk of Bias 2/Risk of Bias in Non-randomized Studies - of Interventions), random-effects pooling, and GRADE were used. In total, 15 studies (4 randomised, 11 observational; >6,000 patients) were included. DOAC exposure was not associated with higher mortality (risk ratio (RR) = 0.98, 95% confidence interval (CI) = 0.66-1.45), transfusion (RR = 1.20, 95% CI = 0.96-1.49), or VTE (RR = 0.41, 95% CI = 0.16-1.05). DOAC users more often experienced operative delay beyond 24-36 hours (RR = 2.43, 95% CI = 0.93-6.33), which may reflect institutional caution. Evidence certainty ranged from low to moderate. In orthopaedic trauma, DOAC use is not linked to increased perioperative bleeding, transfusion, VTE, or mortality, but is associated with modest surgical delay. Where renal function is adequate and no active bleeding exists, early fixation without routine pharmacological reversal appears safe. Standardised early-surgery pathways and education on DOAC pharmacokinetics may reduce avoidable delays.

## Introduction and background

Direct oral anticoagulants (DOACs; apixaban, rivaroxaban, edoxaban, dabigatran) are widely used for the prophylaxis and treatment of thromboembolic disease, including atrial fibrillation and both arterial and venous thrombosis, with meta-analyses showing favourable profiles versus warfarin and simpler perioperative logistics than vitamin K antagonists (VKAs), yet perioperative management in urgent orthopaedic trauma remains inconsistent [1]. Timely fixation for hip fracture and other time-critical trauma (≈24-48 hours) reduces mortality and complications and is embedded in guidelines, but concerns about residual DOAC effect, especially with renal impairment, can conflict with early surgery [2-4]. Although specific reversal agents exist (idarucizumab, andexanet alfa), their use is limited by cost, access, and uncertain impact in fracture care; hence, many centres adopt holding strategies rather than reversal [5,6]. Recent reviews in hip fracture cohorts highlight frequent delays to theatre without consistent increases in perioperative bleeding or transfusion, albeit from heterogeneous, largely observational evidence [7,8]. Observational series echo this: some report DOAC-related delays beyond local targets, while others show comparable short-term outcomes after adjustment [9-11]. Meta-analyses across oral anticoagulants also suggest longer time to surgery and higher mortality versus non-anticoagulated patients, underscoring the need to isolate DOAC-specific effects [12].

The objective of this review was to address two related but distinct clinical questions: (1) among adults presenting with orthopaedic trauma already receiving a DOAC, how do perioperative outcomes (bleeding/transfusion, time to surgery, mortality, and length of stay) compare with patients on VKAs or not receiving anticoagulation; and (2) in adults undergoing operative trauma care, how does postoperative DOAC thromboprophylaxis compare with low-molecular-weight heparin (LMWH) in preventing symptomatic venous thromboembolism (VTE)?

## Review

Methodology

Search Strategy

We systematically searched MEDLINE (PubMed), Embase (Ovid), and Cochrane CENTRAL for English-language human studies published between January 2010 and 1 October 2025. This period reflects the introduction and routine clinical use of DOACs. Controlled vocabulary and free-text terms encompassed DOAC agents, orthopaedic trauma and fractures, operative care, bleeding outcomes, and venous thromboembolism. No filters for study design, outcomes, or publication type were applied to minimise unintended exclusions. Full search strategies for each database are provided in the Appendices. Records were imported into Rayyan to facilitate de-duplication and screening [13].

Study Selection

Study selection followed prespecified eligibility criteria. Two reviewers independently screened all titles and abstracts, followed by full-text assessment of potentially eligible articles. Discrepancies were resolved by consensus, with recourse to a third reviewer when needed. The process adhered to the Preferred Reporting Items for Systematic Reviews and Meta-Analyses (PRISMA) 2020 guidance [14]. This review was not prospectively registered, as data extraction and preliminary analyses had been completed before the decision to formalise the review protocol. The review was not registered in PROSPERO because the project had progressed beyond data extraction at the time registration was considered, which does not meet PROSPERO’s eligibility requirements.

Eligibility Criteria

Studies were eligible if they involved (1) adult orthopaedic trauma patients receiving any DOAC (apixaban, rivaroxaban, dabigatran, edoxaban, or betrixaban) either preoperatively (ongoing treatment at the time of injury) or postoperatively (initiated for thromboprophylaxis); and (2) reported at least one of the following outcomes: perioperative bleeding or transfusion, mortality, time to surgery or operative delay, length of stay, or VTE (deep vein thrombosis (DVT)/pulmonary embolism (PE)). Preoperative exposure studies contributed to analyses of mortality, bleeding/transfusion, time to surgery, and length of stay, whereas postoperative prophylaxis trials informed the VTE outcome meta-analysis. Eligible designs included randomised controlled trials, prospective or retrospective cohort studies, and case-control studies. Studies were excluded if they (1) exclusively examined elective arthroplasty or non-orthopaedic surgery; (2) included mixed surgical cohorts where trauma data were inseparable; or (3) represented case reports, animal, cadaveric, or paediatric data. We prespecified two distinct questions and analysed them separately: (1) pre-injury DOAC exposure and perioperative outcomes (mortality, transfusion, operative delay), based on observational cohorts; and (2) postoperative DOAC prophylaxis versus LMWH for VTE prevention, based on randomised and comparative studies. No meta-analysis pooled studies across these two questions.

Outcomes and Definitions

We prespecified all outcomes for this review and grouped them according to the relevant clinical context. For the pre-injury DOAC exposure question, the primary perioperative outcomes were mortality, transfusion, bleeding, operative delay, and length of stay. Mortality was recorded as in-hospital or 30-day mortality, depending on how individual studies reported it. Transfusion was defined as receipt of red blood cell units within the perioperative period, generally within the first 48-72 hours after surgery, following each study’s reported thresholds. Bleeding comprised major or clinically relevant non-major bleeding, using the definitions provided by the individual cohorts. Operative delay reflected time to surgery exceeding the threshold specified in the source study, most commonly more than 24, 36, or 48 hours after presentation or last DOAC dose. Length of stay was extracted as the total acute hospital stay in days. For the postoperative thromboprophylaxis question, the principal outcome was VTE. This included symptomatic DVT or PE confirmed through imaging or clinical evaluation as defined by the trial protocols. Postoperative bleeding outcomes were extracted using the major and clinically relevant non-major bleeding criteria employed in each study. Because definitions varied across observational studies and randomised trials, outcomes were extracted exactly as reported by the original authors, and the review adhered to these definitions to avoid introducing additional classification bias.

Data Extraction and Risk of Bias

Two reviewers independently extracted study characteristics, populations, interventions/comparators, and outcomes (mortality, bleeding/transfusion, time to surgery, VTE, length of stay). Percentages were converted to counts when denominators were available. Randomised trials were assessed using the Risk of Bias 2 (RoB 2) [15] and observational studies the the Risk of Bias in Non-randomised Studies - of Interventions (ROBINS-I) tools [16]; disagreements were resolved by discussion. Visualisation of domain-level and overall risk of bias judgments was undertaken using the robvis web application [17] (available at https://www.riskofbias.info/welcome/robvis-visualization-tool).

Data Synthesis and Statistical Analysis

When ≥3 studies reported comparable outcomes, we pooled risk ratios (RRs) for dichotomous outcomes and mean differences (MDs) for continuous outcomes using random-effects DerSimonian-Laird models. Adjusted effect estimates (adjusted odds ratio (aOR), adjusted risk ratio (aRR), adjusted hazard ratio (aHR)) were extracted and synthesised using generic inverse-variance random-effects models whenever available. When adjusted data were unavailable, unadjusted event-count pooling was undertaken as an exploratory analysis and interpreted cautiously because of expected confounding in observational designs. Heterogeneity was evaluated using I², τ², and Cochran’s Q, supplemented by leave-one-out influence analysis, stratified subgroup analyses (study design, comparator type, hip-only versus mixed trauma, perioperative practice patterns), and prediction intervals for outcomes with substantial heterogeneity (I² > 75%). Medians and interquartile ranges were converted to means and standard deviations using the transformation functions embedded within the meta and metafor packages as implemented in the MetaAnalysisOnline platform, which also applies the standard 0.5 continuity correction for zero-event arms [18-20]. Because several comparisons involved few studies and sparse events, we retained the prespecified random-effects DerSimonian-Laird models with the platform’s default continuity correction; these unadjusted syntheses are designated exploratory and interpreted cautiously. Publication bias assessment was not performed because fewer than 10 studies contributed to each meta-analysis. Analyses used MetaAnalysisOnline [18], which employs R [19] using the metafor [20] and meta [21] packages; narrative synthesis was used where pooling was inappropriate. Certainty of evidence was graded using GRADE [22].

Results

Study Selection

**Figure 1:**
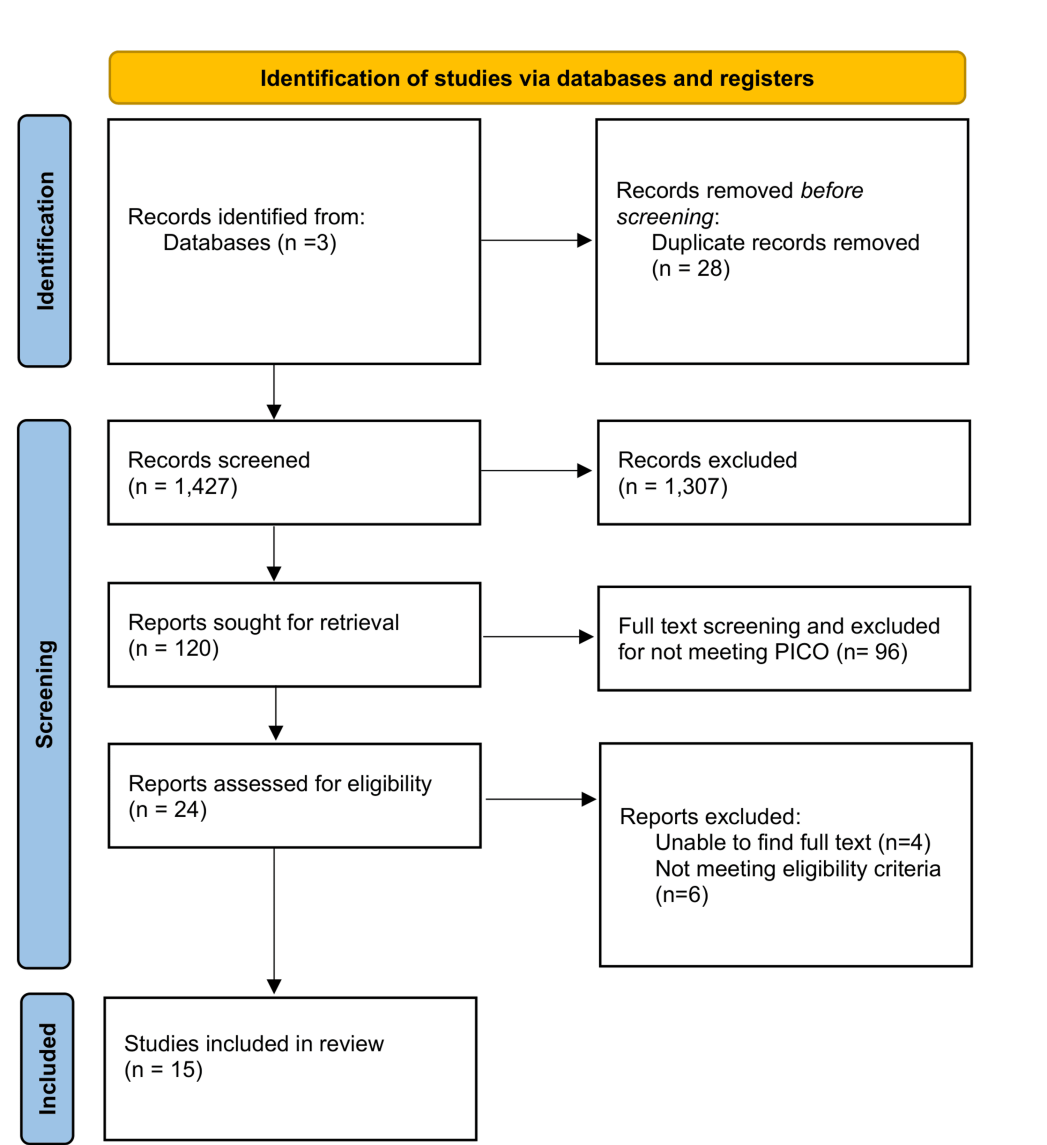
Preferred Reporting Items for Systematic Reviews and Meta-Analyses (2020) flow diagram illustrating the study selection process. Adapted from Page et al. [13], under a Creative Commons Attribution License (CC BY 4.0).

The search retrieved 1,455 records. After removing 28 duplicates, 1,427 unique citations were screened. Full-text review was performed for 120 articles. Of these, 105 were excluded (reasons summarised in the Appendices), leaving 15 studies that met the inclusion criteria and were included in the qualitative and quantitative syntheses. Although the search extended to October 2025, no additional eligible studies were identified from 2023 to 2025. The PRISMA flow diagram (Figure 1) summarises the selection process.

Study Characteristics

In total, 15 studies published between 2014 and 2022 were included in this systematic review, comprising four randomised controlled trials and eleven observational cohort or registry studies (Table 1). Sample sizes ranged widely, from fewer than 50 to over 2,000 participants, reflecting both single-centre case-control series and large national registry analyses. The studies were conducted across Asia, Europe, Australia, and North America, encompassing a representative global distribution of perioperative orthopaedic trauma populations. Studies such as Fuji et al. (2014) [23], Tang et al. (2017) [24], Campbell et al. (2017), Goh et al. (2020) [25], and John et al. (2022) [26] evaluated postoperative thromboprophylaxis using DOACs such as edoxaban, rivaroxaban, or apixaban, compared with LMWH. The remaining observational studies primarily examined patients presenting with hip fractures while receiving DOACs pre-injury, comparing perioperative outcomes against those on warfarin, antiplatelet agents, or without anticoagulation. Across studies, the most frequently assessed outcomes included perioperative mortality, bleeding or transfusion requirements, time to surgery, and thromboembolic events. A minority of studies also reported the length of hospital stay or other postoperative complications. While most observational series employed institutional early-surgery protocols without pharmacological reversal, two large registry studies, Daugaard et al. and Hoerlyck et al. [10,27], contributed adjusted national-level estimates. Overall, the included literature reflects a mix of real-world and experimental data addressing both prophylactic use of DOACs and their perioperative management in orthopaedic trauma. Most studies focused on hip fracture surgery, though two included broader trauma populations; hip fracture-only subgroup analyses are presented separately where data permitted.

**Table 1:** Characteristics of the included studies (n = 15). AC = anti-coagulation; ATT = antithrombotic therapy; ASA = acetylsalicylic acid; AE = adverse events; BNF = British National Formulary; COU = coumadin; CRIF = closed reduction internal fixation; DOAC = direct oral anticoagulant; DVT = deep vein thrombosis; Hb = haemoglobin; PAI = platelet aggregation inhibitors; PCC = prothrombin complex concentrate; PE = pulmonary embolism; VKA = vitamin K antagonist; THA = total hip arthroplasty; LMWH = low-molecular-weight heparin; VTE = venous thromboembolism; LOS = length of stay: TTS = time to surgery

Study (first author, year)	Country	Study design	Sample size (total/DOAC)	DOAC(s) studied	Comparator(s)	Surgical population/procedure	Key peri-operative strategy	Primary outcomes reported
Fuji et al., 2014 [23]	Japan	Multicenter, randomised, open-label, phase 3 trial	92/62	Edoxaban 30 mg once daily	Enoxaparin 2,000 IU SC twice daily	Hip fracture surgery (femoral neck, trochanteric, subtrochanteric)	Postoperative thromboprophylaxis with edoxaban vs. enoxaparin for 11–14 days, started 6–24 hours post-surgery	Major/Clinically relevant non-major bleeding, any bleeding, thromboembolic events (DVT/PE/mortality)
Campbell et al., 2017 [28]	United States	Retrospective cohort (national database)	4,090/929	Factor Xa inhibitors (class)	Enoxaparin (n = 2,326), VKA (n = 835)	Hip fracture surgery (fixation; THA excluded)	Postoperative pharmacologic prophylaxis within 2 weeks	DVT, PE, bleeding events, anaemia, and transfusion rates
Li et al., 2017 [29]	China	Prospective randomised controlled trial	80/39	Rivaroxaban 10 mg daily (pre-op)	Conservative (no pre-op pharmacologic prophylaxis)	Femoral neck fracture (THA or hemiarthroplasty)	Preoperative rivaroxaban on admission until 1 day pre-surgery; all received post-op rivaroxaban	Pre- and postoperative DVT/PE incidence, major bleeding
Tang et al., 2017 [24]	China	Prospective double-blind RCT	287/96	Rivaroxaban 10 mg daily	Enoxaparin 4,000 IU SC daily; sequential enoxaparin → rivaroxaban	Internal fixation for hip fracture	Compared 3 regimens: (1) rivaroxaban × 28 days; (2) enoxaparin × 28 days; (3) enoxaparin 1 week → rivaroxaban to day 28	VTE incidence (DVT/PE), bleeding, wound issues, compliance, treatment cost
Franklin et al., 2018 [30]	United States	Multicentre retrospective case–control	93/19	Dabigatran, apixaban, rivaroxaban	74 matched controls (no anticoagulation)	Geriatric proximal femur fractures (CMN, SHS, hemiarthroplasty)	Early surgery (<48 hours) on DOAC vs. controls; no reversal agents	Time-to-surgery, EBL, transfusion, Hb change, wound issues, readmission, 30/90-day and 1-year survival
Mullins et al., 2018 [31]	United Kingdom	Retrospective case–control	125/63	Rivaroxaban, apixaban, dabigatran	62 matched controls (no anticoagulation)	Hip fracture (hemiarthroplasty, SHS, IM nail, THA)	Early surgery without DOAC washout; no reversal	Hb change, transfusion, wound complications, 30-day mortality
Daugaard et al., 2019 [27]	Denmark	Nationwide population-based cohort	74,791 total; 1,063 DOAC users	Dabigatran, rivaroxaban, apixaban, edoxaban	Non-users; VKA; antiplatelet users	All hip fracture surgeries	Observational; adjusted by comorbidity and surgical delay	RBC transfusion < 7 days; 30-day all-cause mortality
Bruckbauer et al., 2019 [32]	Austria	Retrospective cohort	320 total; DOAC 54	Dabigatran (13), rivaroxaban (34), apixaban (7)	VKA (59), no-ATT (207)	Isolated hip fractures ≥ 65 y	VKA → vit K ± PCC; DOAC → idarucizumab (1) or PCC (1); DOAC = longest surgical delay	DOAC longest TTS; transfusion ≈ COU; no ↑ bleeding/ICU LOS/mortality vs. COU
Hourston et al., 2020 [9]	UK	Retrospective cohort	844 total; DOAC 32; VKA83; no-AC 729	Rivaroxaban (19), apixaban (8), dabigatran (5)	VKA; no anticoagulation	Neck of femur fractures (fixation, hemiarthroplasty, THA)	Standard care; VKA reversal (Vit K ± PCC); no DOAC antidote	Anticoag ↑ TTS (p = 0.028); DOAC → delay > 36 hours (p = 0.001) but not > 48 hours; no ↑ LOS; 30-day survival ↓ with VKA, not DOAC
Schermann et al., 2019 [11]	Israel	Retrospective cohort (2011–2016)	CRIF 1,143/60 DOAC; HA 571/29 DOAC (Total 89)	Apixaban, rivaroxaban, dabigatran	No-AC (primary)	Proximal hip fractures > 65 y (CRIF/HA)	Operate 24–36 hours after last DOAC (apixaban/ rivaroxaban), 12–24 hours (dabigatran); no reversal	No ↑ bleeding/transfusion; DOAC–CRIF → longer delay, ↑ 1-year mortality unadjusted; delay drives risk; advocate earlier surgery (~12 hours)
Goh et al., 2020 [25]	UK	Retrospective cohort	321/54 DOAC	Apixaban (80%), rivaroxaban (17%), dabigatran (4%)	LMWH (dalteparin)	Older adults hip fracture	Postoperative VTE prophylaxis 6 weeks; DOAC vs. LMWH per BNF guidelines	VTE 0% DOAC vs. 3.4% LMWH (NS); Hb 7.4% vs. 3.0% (NS); transfusion and mortality similar
King et al., 2020 [33]	Australia	Retrospective cohort	84 total (DOAC early <48 hours 17; delayed >48 hours 11)	Apixaban, dabigatran, rivaroxaban	Matched non-DOAC controls	Neck of femur fractures	Compared early (<48 hours) vs. delayed (>48 hours) surgery on DOAC	Hb loss and transfusion no diff.; 90-day mortality 0% early vs. 36% delayed (p = 0.04); DOAC not a reason to delay
Hoerlyck et al., 2019 [10]	UK/Denmark	Cross-sectional registry	2307 total; DOAC 33	Rivaroxaban, apixaban	VKA; no-AC	Hip fracture surgery (≥50 years)	VKA → vit K (INR < 1.5); DOAC > 24 hours after last dose; AC resumed day 1 postoperatively (bridge LMWH for VKA)	TTS 27 hours (AC) vs. 25 hours (no AC); no diff. mortality, transfusion, LOS after adjustment; safe without delay
Schuetze et al., 2019 [34]	Germany	Retrospective chart review	327 total; DOAC 52 (VKA 25; ASA 74; PAI 30; none 146)	Rivaroxaban, dabigatran, apixaban	No-AC; VKA; antiplatelets	Inter/subtrochanteric hip	ASA/PAI continued; VKA reversed (vit K/PCC); DOAC/VKA bridged with heparin and restarted day 7	DOAC → 3.4× ↑ transfusion risk; Hb drop similar; no ↑ complications or mortality; early fixation safe
John et al., 2022 [26]	USA	Prospective RCT (Level II)	121/58 DOAC	Rivaroxaban 10 mg × 20 days → ASA × 3 weeks	Enoxaparin 40 mg × 20 days → ASA × 3 weeks	Operative orthopaedic trauma (pelvis/lower extremity)	Randomised at discharge; standard LMWH inpatient; no routine VTE screen	Rivaroxaban ↑ patient satisfaction; adherence same; no VTE/bleed difference; fewer AEs; cheaper

Risk of Bias Within Studies

Risk of bias was assessed using the Cochrane RoB 2 tool [15] for randomised controlled trials and the ROBINS-I tool [16] for non-randomised studies. Among the randomised studies, Fuji et al. (2014) [23] and Li et al. (2017) [29] demonstrated some concerns overall, largely due to unclear allocation concealment and limited reporting of prespecified protocols, though both maintained low risk for missing data and deviations from intended interventions. The Tang et al. (2017) [24] study was rated low risk of bias across all domains except for minor uncertainty regarding protocol availability. The John et al. (2022) [26] study was judged high risk of bias, primarily because of unblinded assessment and subjective, patient-reported outcome measures, despite otherwise low risk in randomisation and follow-up domains.

For the non-randomised studies, risk of bias ranged from moderate to serious across most domains, driven chiefly by confounding and selection bias. Studies such as Campbell et al. (2017) [28], Franklin et al. (2018) [30], Mullins et al. (2018) [31], Bruckbauer et al. (2019) [32], Hourston et al. (2020) [9], Schermann et al. (2019) [11], Goh et al. (2020) [25], King et al. (2020) [33], Hoerlyck et al. (2019) [10], and Schuetze et al. (2019) [34] were considered to have serious overall risk of bias, mainly reflecting incomplete adjustment for baseline differences, surgical timing, and anticoagulant indication. Only Daugaard et al. (2019) [27] achieved a moderate overall risk of bias due to robust registry-based adjustment for surgical delay.

**Figure 2:**
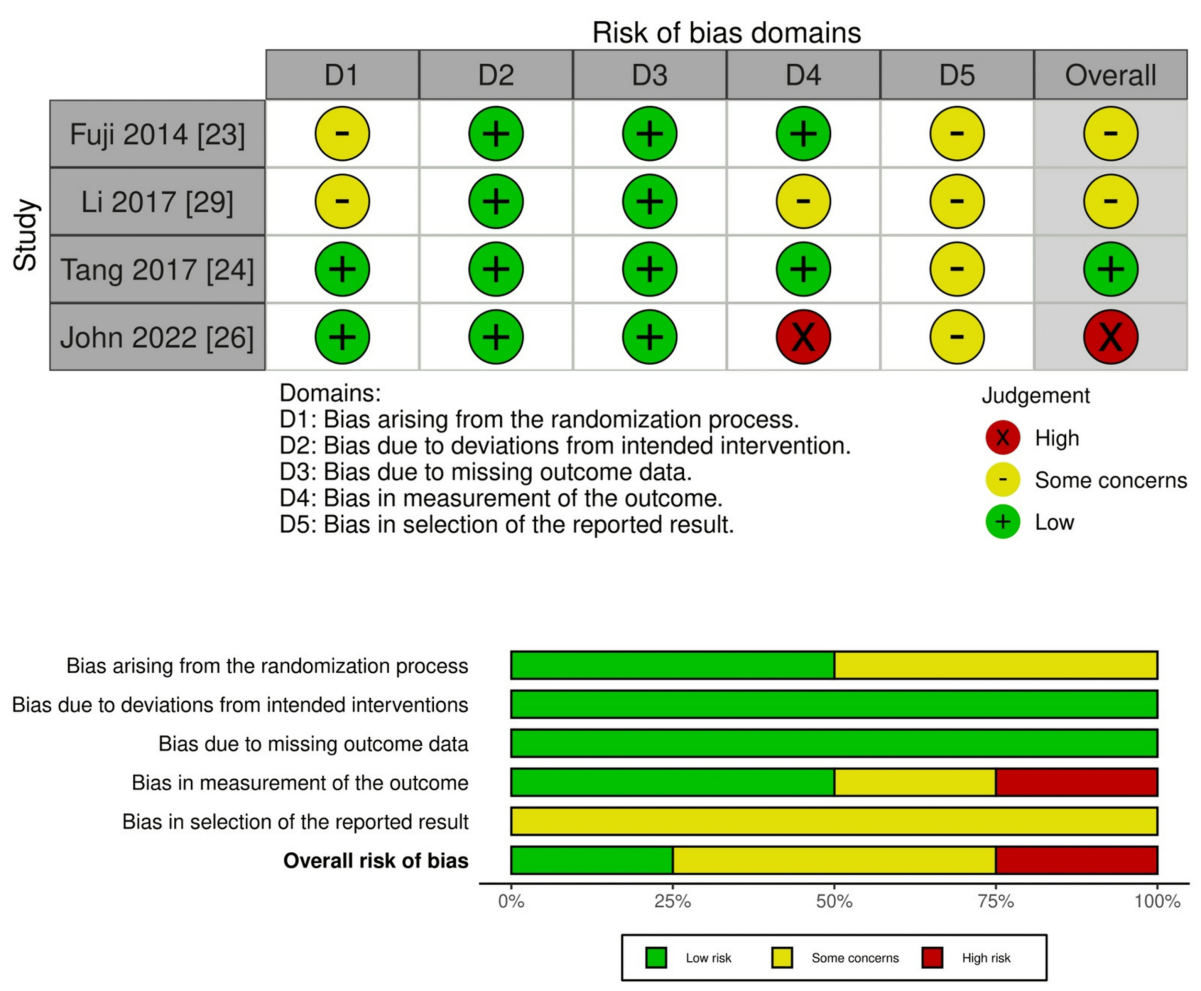
Risk of bias assessment for randomised controlled trials (n = 4). Traffic-light plot (upper panel) and summary bar plot (lower panel) showing domain-level and overall risk-of-bias judgments for the four randomised controlled trials. RoB 2 domains: D1 = bias arising from the randomisation process; D2 = bias due to deviations from intended interventions; D3 = bias due to missing outcome data; D4 = bias in measurement of the outcome; D5 = bias in selection of the reported result. Green = low risk, yellow = some concerns. Credits: Risk of bias was assessed using the RoB 2 tool [15], and plots were visualised with robvis [17].

**Figure 3:**
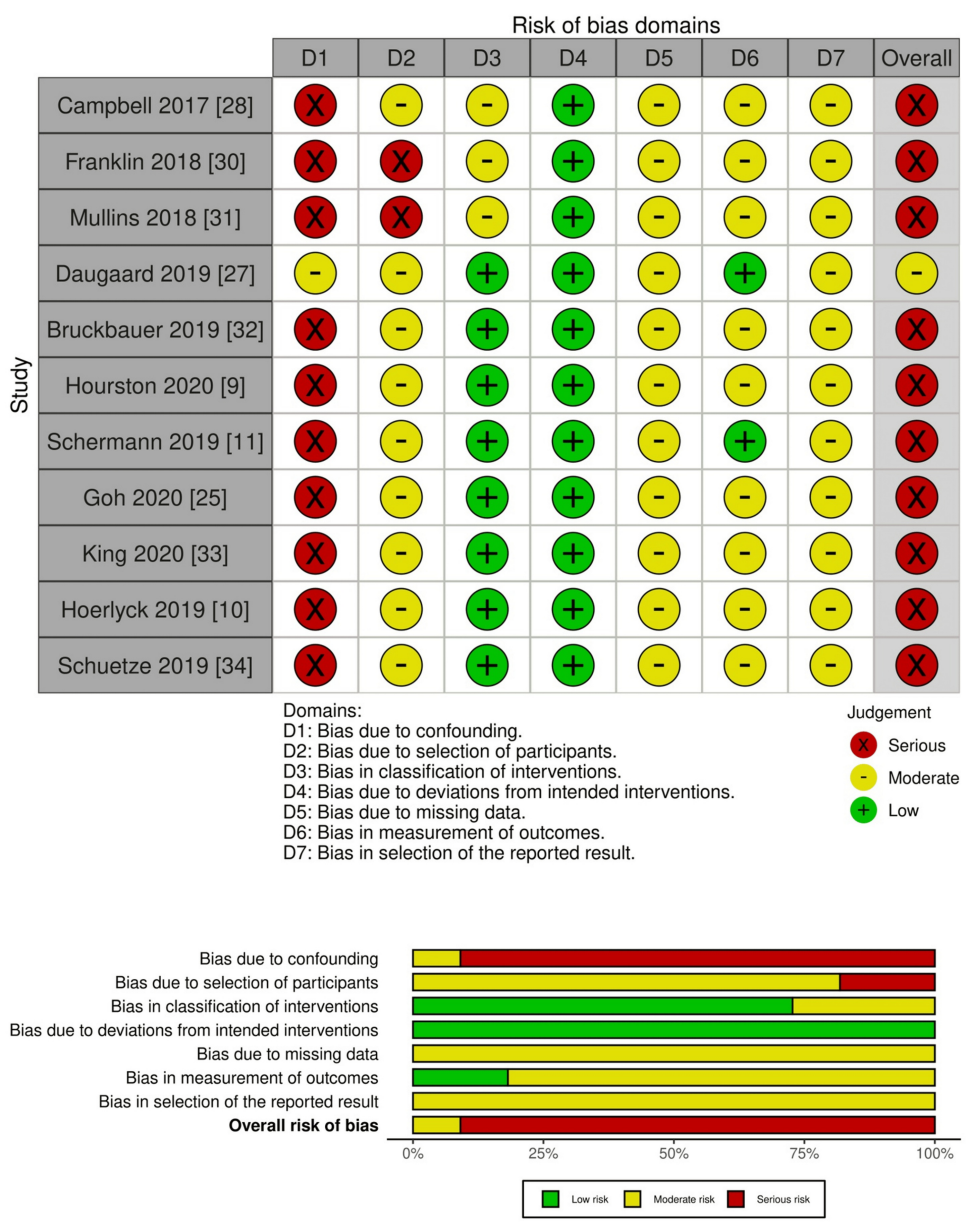
Risk of bias assessment for non-randomised studies (n = 11). Traffic-light plot (upper panel) and summary bar plot (lower panel) showing domain-level and overall risk-of-bias judgments for the 11 non-randomised observational studies. ROBINS-I domains: D1 = bias due to confounding; D2 = bias due to selection of participants; D3 = bias in classification of interventions; D4 = bias due to deviations from intended interventions; D5 = bias due to missing data; D6 = bias in measurement of outcomes; D7 = bias in selection of the reported result. Green = low risk, yellow = moderate risk, red = serious risk. Credits: Risk of bias was assessed using ROBINS-I [16], and plots were visualised with robvis [17].

Overall, the randomised evidence was of low-to-moderate quality, while the non-randomised evidence was generally moderate-to-serious risk of bias, as visualised in Figure 2 and Figure 3, where confounding and reporting limitations were the predominant sources of potential bias.

Quantitative Synthesis (Meta-Analysis)

**Figure 4:**
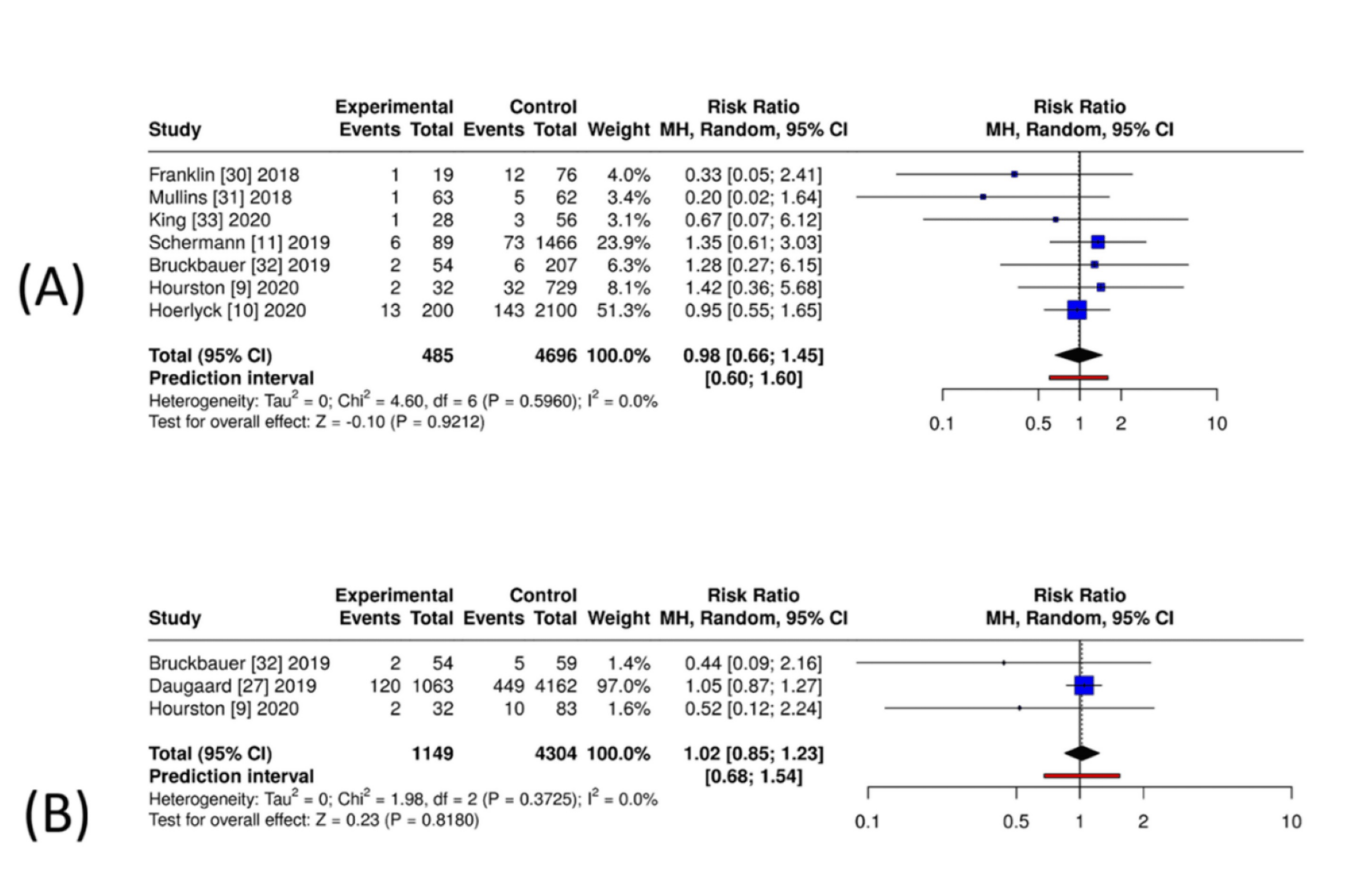
Exploratory unadjusted pooled estimates for perioperative mortality in orthopaedic trauma patients exposed to DOACs. (A) DOAC versus no anticoagulation: exploratory unadjusted pooled RRs and 95% confidence intervals from seven observational cohorts. (B) DOAC versus VKAs: exploratory unadjusted pooled RRs from three observational cohorts. Analyses used random-effects models applied to unadjusted event-count data and are interpreted cautiously due to expected confounding in observational designs. Produced using MetaAnalysisOnline [18]. DOAC = direct oral anticoagulant; VKA = vitamin K antagonist; RR = risk ratio

Mortality: Across seven comparative cohorts (DOAC vs. no anticoagulation; n = 485 DOAC, n = 4,696 controls), the pooled effect showed no difference in 30-day/in-hospital mortality (RR = 0.98, 95% CI = 0.66-1.45; I² = 0%) (Figure 4A). Against VKAs (three studies; n = 1,149 DOAC, n = 4,304 VKA), there was likewise no difference (RR = 1.02, 95% CI = 0.85-1.23; I² = 0%) (Figure 4B). As only one study assessed mortality comparing DOAC users with a mixed non-DOAC comparator cohort, no meta-analysis was performed. The single large registry study reported a similar mortality risk between groups (RR = 1.08, 95% CI = 0.91-1.27). Given the observational design and residual confounding, these findings should be interpreted cautiously.

**Figure 5:**
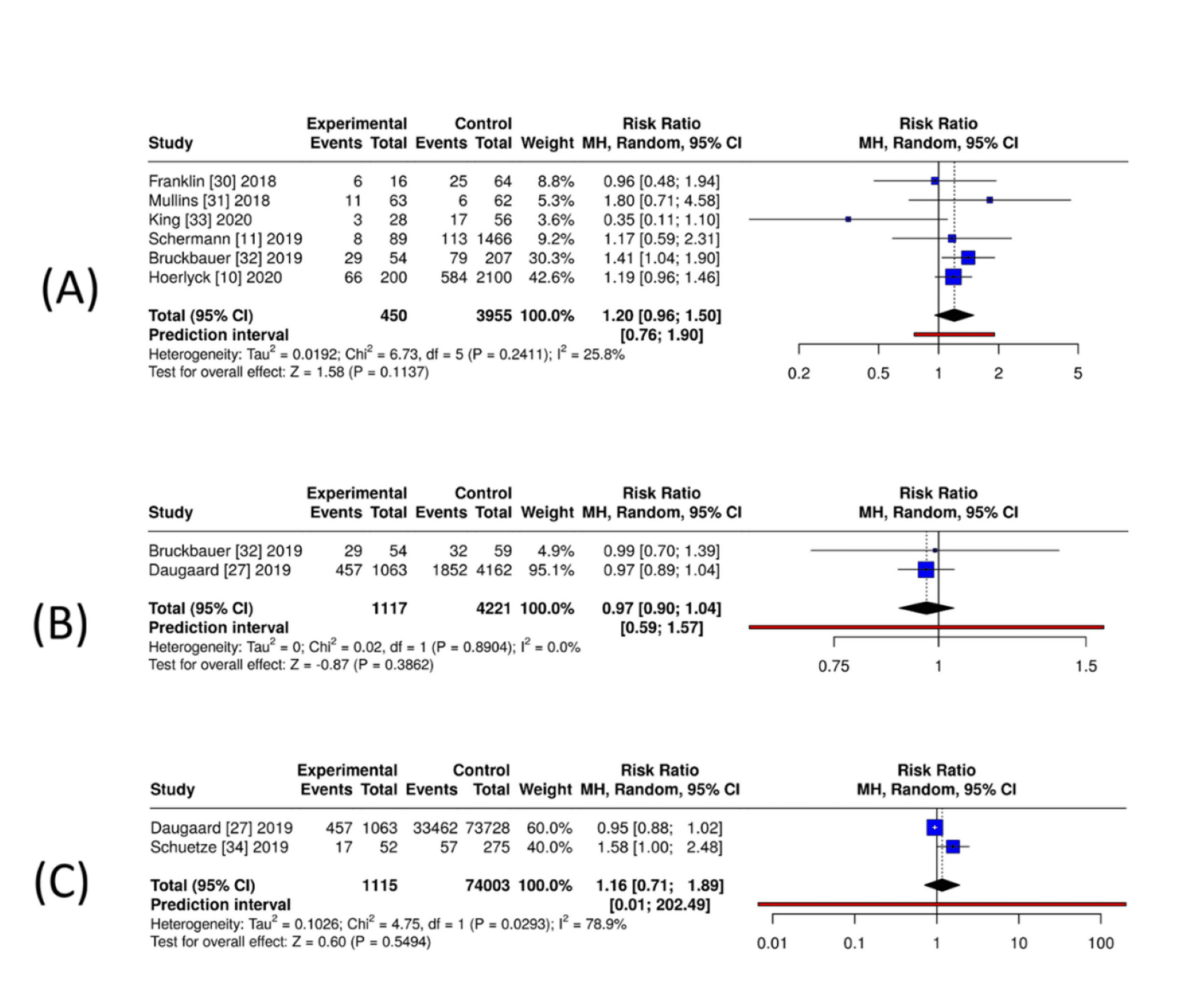
Exploratory unadjusted pooled estimates for perioperative transfusion in patients exposed to DOACs. (A) DOAC versus no anticoagulation: exploratory unadjusted pooled RRs from six observational cohorts. (B) DOAC versus VKA: exploratory unadjusted pooled RRs from two observational cohorts. (C) DOAC versus mixed comparator groups: exploratory unadjusted pooled RRs from two heterogeneous studies, with substantial between-study variation. All analyses used random-effects models on unadjusted data; findings are interpreted cautiously because of confounding and, for panel C, high heterogeneity. Generated using MetaAnalysisOnline [18]. DOAC = direct oral anticoagulant; VKA = vitamin K antagonist; RR = risk ratio

Transfusion requirements: Six studies comparing DOAC with no anticoagulation (n = 450 DOAC, n = 3,955 controls) showed a non-significant trend toward higher transfusion with DOAC (RR = 1.20, 95% CI = 0.96-1.50; I² = 25.8%) (Figure 5A). Two studies comparing DOAC with VKA (n = 1,117 DOAC, n = 4,221 VKA) showed no difference (RR = 0.97, 95% CI = 0.90-1.04; I² = 0%) (Figure 5B). For DOAC vs. mixed comparators (two studies; n = 1,115 DOAC, n = 74,003 controls), the pooled estimate was imprecise and heterogeneous (RR = 1.16, 95% CI = 0.71-1.89; I² = 79%) (Figure 5C), driven by the small single-centre series paired with the large registry. DOAC users did not require more transfusions than VKA users, and any apparent increase versus non-anticoagulated patients was small and not statistically significant after pooling.

**Figure 6:**
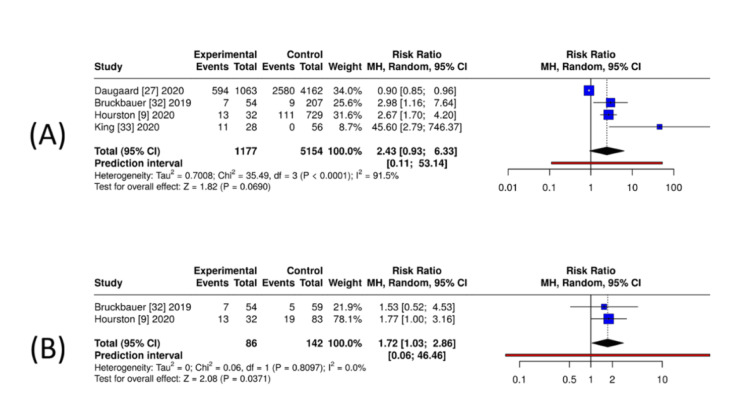
Exploratory unadjusted pooled estimates for operative delay (>24-36 hours) among DOAC-exposed orthopaedic trauma patients. (A) DOAC versus no anticoagulation: exploratory unadjusted pooled RRs from four observational cohorts, with substantial heterogeneity (I² > 90%). (B) DOAC versus VKA: exploratory unadjusted pooled RRs from two observational cohorts. Analyses used random-effects models based on unadjusted event-count data; confounding and high heterogeneity limit causal interpretation. Produced using MetaAnalysisOnline [18]. DOAC = direct oral anticoagulant; VKA = vitamin K antagonist; RR = risk ratio

Operative delay (>24-36-hour thresholds): Four studies directly comparable for DOAC versus no anticoagulation (n = 1,177 DOAC, n = 5,154 controls) showed a higher frequency of delay among DOAC users (RR = 2.43, 95% CI = 0.93-6.33; I² = 91.5%), although the estimates were imprecise and heterogeneous (Figure 6A). Two studies comparing DOAC versus VKA (n = 86 DOAC, n = 142 VKA) suggested a higher risk of delay in DOAC (RR = 1.72, 95% CI = 1.03-2.86; I² = 0%) (Figure 6B). Only one registry study reported operative delay for DOAC users versus a mixed comparator group, so no pooled analysis was performed. That study found higher unadjusted rates of delay among DOAC users (RR = 1.42, 95% CI = 1.35-1.50), although adjustment reduced the effect. DOAC exposure is associated with a moderate increase in early-surgery delay versus non-anticoagulated patients, but timing is comparable to warfarin users, and delays did not translate into worse clinical outcomes in pooled analyses.

**Figure 7:**
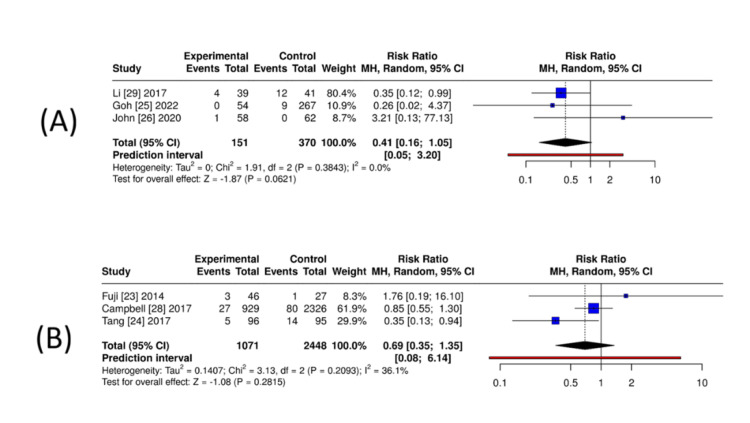
Pooled estimates for symptomatic VTE in orthopaedic trauma or postoperative patients receiving DOACs. (A) DOAC versus no anticoagulation: exploratory unadjusted pooled RRs from three small observational cohorts. (B) DOAC versus enoxaparin (LMWH prophylaxis): pooled RRs from three randomised/comparative studies; analyses used inverse-variance random-effects synthesis where adjusted estimates were available. Panel A represents exploratory unadjusted analyses with expected confounding; panel B reflects higher-certainty RCT-driven estimates. Generated using MetaAnalysisOnline [18]. VTE = venous thromboembolism; DOAC = direct oral anticoagulant; LMWH = low-molecular-weight heparin; RR = risk ratio; RCT = randomised controlled trial

VTE (symptomatic DVT/PE): Three studies comparing DOAC with no anticoagulation (n = 151 DOACs, n = 370 controls) showed no increase in VTE and a trend toward fewer events with DOAC (RR = 0.41, 95% CI = 0.16-1.05; I² = 0%) (Figure 7A). When DOAC was compared with enoxaparin for prophylaxis (three studies; n = 1,071 DOAC, n = 2,448 enoxaparin), pooled effects showed no significant difference (RR = 0.69, 95% CI = 0.35-1.35; I² = 36%) (Figure 7B). Only one study compared VTE rates between DOAC and VKA users; therefore, no meta-analysis was performed. That study reported a lower risk of VTE in DOAC users (RR = 0.58, 95% CI = 0.36-0.93). Across trauma cohorts and prophylaxis trials, VTE risk with DOAC is comparable to, or lower than, comparators.

Summary of Findings and Certainty of Evidence

The overall strength and certainty of evidence for each pooled outcome, evaluated using the GRADE framework, are summarised in Table 2.

**Table 2:** Summary of findings and GRADE assessment for key outcomes. Footnotes: a. Downgraded one level for serious risk of bias due to confounding in observational studies contributing to the estimate. b. Downgraded one level for inconsistency (substantial heterogeneity, I² > 50%). c. Downgraded one level for imprecision due to wide confidence intervals crossing the line of no effect. d. Downgraded for indirectness because VTE data included prophylaxis RCTs in postoperative patients rather than pre-injury DOAC trauma cohorts. e. No upgrading criteria met (no large effect, dose–response, or residual confounding likely to reduce the effect). Legend: Certainty ratings follow the GRADE Working Group approach (high ⬤⬤⬤⬤, moderate ⬤⬤⬤◯, low ⬤⬤◯◯, very low ⬤◯◯◯). Randomised trials start as high certainty and may be downgraded for bias, inconsistency, or imprecision. Observational studies start as low certainty but may be upgraded for consistency, large effect, or directness. “Absolute effect” indicates direction of difference, not absolute risk difference (due to heterogeneity of baseline event rates). DOAC = direct oral anticoagulant; RCT = randomised controlled trial; RR = risk ratio; CI = confidence interval Credits: GRADE approach adapted from GRADE Working Group guidance [22].

Outcome and comparator	Number of studies (design)	Participants (DOAC/Control)	Relative effect (RR, 95% CI)	I² (%)	Absolute effect	Certainty (GRADE)
Mortality — DOAC vs. None	7 (obs)	485/4,696	0.98 (0.66–1.45)	0	No difference	⬤⬤◯◯ Low–Moderate
Mortality — DOAC vs. VKA	3 (obs; registry + cohorts)	1,149/4,304	1.02 (0.85–1.23)	0	No difference	⬤⬤◯◯ Low–Moderate
Mortality — DOAC vs. Mixed	1 (obs; registry)	1,063/73,728	1.08 (0.91–1.27)	—	No difference	⬤◯◯◯ Very low
Transfusion — DOAC vs. None	6 (obs)	450/3,955	1.20 (0.96–1.50)	25.8	No difference	⬤⬤◯◯ Low
Transfusion — DOAC vs. VKA	2 (obs)	1,117/4,221	0.97 (0.90–1.04)	0	No difference	⬤⬤◯◯ Low
Transfusion — DOAC vs. Mixed	2 (obs)	1,115/74,003	1.16 (0.71–1.89)	79	No difference	⬤◯◯◯ Very low
Operative delay >24–36 hours — DOAC vs. None	4 (obs)	1,177/5,154	2.43 (0.93–6.33)	91	↑ Increased delay	⬤⬤◯◯ Low
Operative delay — DOAC vs. VKA	2 (obs)	86/142	1.72 (1.03–2.86)	0	No difference	⬤⬤◯◯ Low
Operative delay — DOAC vs. Mixed	1 (obs; registry)	1,063/73,728	1.42 (1.35–1.50)	—	↑ Increased delay (unadjusted)	⬤◯◯◯ Very low
VTE — DOAC vs. None	3 (RCT/obs mix)	151/370	0.41 (0.16–1.05)	0	No difference	⬤⬤⬤◯ Moderate
VTE — DOAC vs. Enoxaparin	3 (2 RCTs + 1 obs)	1,071/2,448	0.69 (0.35–1.35)	36	No difference	⬤⬤⬤◯ Moderate
VTE — DOAC vs. VKA	1 (obs)	929/835	0.58 (0.36–0.93)	—	↓ Lower VTE with DOAC	⬤⬤◯◯ Low

Discussion

This systematic review and meta-analysis synthesised evidence from 15 studies, including four randomised controlled trials and 11 observational cohorts, evaluating perioperative outcomes in orthopaedic trauma patients treated with DOACs. Across pooled analyses encompassing more than 6,000 patients, DOAC exposure was not associated with increased mortality, bleeding, transfusion, or thromboembolic risk, but was linked to a moderate increase in surgical delay beyond 24-36 hours. These findings reinforce the gap between pharmacological safety data and real-world operative timing practices in trauma care. Findings from pre-injury exposure (perioperative safety) should not be conflated with those from postoperative prophylaxis (VTE efficacy). We, therefore, interpret each evidence stream within its own risk window, comparator, and bias structure.

Principal Findings

Quantitative synthesis showed that perioperative transfusion rates were comparable between DOAC and control cohorts (RR = 1.20, 95% CI = 0.96-1.50), while mortality likewise did not differ significantly (RR = 0.98, 95% CI = 0.66-1.45). The odds of surgery delay beyond institutional targets were higher among DOAC users (RR = 2.43; 95% CI = 0.93-6.33; I² = 91.5%), indicating that anticoagulant status still influences scheduling decisions. Thromboembolic events were infrequent and did not differ between DOAC and LMWH (RR = 0.69, 95% CI = 0.35-1.35). Collectively, these results suggest that DOAC use in trauma patients does not increase clinical risk but continues to be associated with procedural caution and delayed surgery.

Context Within Existing Literature

Prior reviews and registry studies have produced mixed conclusions regarding the impact of DOACs on perioperative outcomes. Hoerlyck et al. found no increase in bleeding or mortality among DOAC-treated hip fracture patients, although delays to surgery remained common [10]. Similarly, Schermann et al. demonstrated that urgent fixation under DOAC influence did not increase transfusion or complications, aligning with our pooled estimates [11]. Hourston et al. likewise reported longer time to theatre but no difference in 30-day outcomes, supporting the view that delays are largely protocol-driven rather than pharmacologically required [9]. Our pooled findings complement trial data from Fuji et al., Tang et al., and John et al., which demonstrated non-inferior efficacy and safety of postoperative thromboprophylaxis with DOACs compared to LMWH [23,24,26]. Together, these results highlight the pharmacological predictability of DOACs and their suitability for standardised perioperative management, contrasting with the persistent caution observed in clinical practice.

Clinical Interpretation

The absence of excess bleeding or mortality suggests surgical delay is often precautionary rather than evidence-based. Most included centres adopted empirical hold times (typically 24-48 hours) without laboratory quantification of DOAC activity. These findings are biologically plausible given the predictable pharmacokinetics of DOACs, which have short half-lives, rapid offset, and limited accumulation in patients with normal renal function. Most trauma patients in the included studies had adequate renal clearance, so residual anticoagulant effect at the time of fixation was likely low. This pharmacological profile helps explain why perioperative bleeding, transfusion, and mortality were not increased despite modest delays to surgery. Given the short elimination half-lives of these agents (8-15 hours) and their predictable clearance in patients with normal renal function, such delays may be unnecessary for the majority of trauma cases. Importantly, studies performing early surgery without pharmacological reversal reported similar transfusion rates and short-term outcomes, suggesting that prompt fixation can be achieved safely when haemostasis and renal function are adequate. The observed delay (RR = 2.43) likely reflects institutional caution and limited familiarity with reversal agents such as idarucizumab and andexanet alfa, which remain expensive and variably available [5,6]. As these agents become more accessible and perioperative confidence grows, practice patterns may better align with current evidence.

Strengths and limitations

This review represents, to our knowledge, the most focused synthesis to date examining DOACs exclusively within trauma populations, excluding elective arthroplasty studies that previously confounded outcomes. Strengths include adherence to PRISMA 2020 guidelines [14], dual independent screening, and validated risk-of-bias assessment (RoB 2 and ROBINS-I) [15,16]. However, several limitations must be acknowledged. Residual confounding from variables such as age, sex, ASA class, Charlson Comorbidity Index, renal function, fracture type, and surgical delay likely persists in observational data. Because several observational studies reported only unadjusted event counts, pooled estimates for these outcomes should be interpreted as exploratory, as residual confounding may have influenced the observed effect sizes. Given the small number of studies and sparse events in several comparisons, we retained the prespecified DerSimonian-Laird random-effects models with continuity corrections, and these analyses should be viewed as exploratory rather than confirmatory. Where reported, renal impairment (estimated glomerular filtration rate <50 mL/minute) was associated with greater delay and bleeding risk, though data were sparse. Few studies reported the precise timing of the last DOAC dose or reversal agent use, precluding subgroup meta-analysis. Most included studies were observational and thus subject to confounding and selection bias. Definitions of “delay” and “bleeding” varied across institutions, contributing to moderate heterogeneity (I² ≈ 50% for timing outcomes). Few studies adjusted for frailty, renal function, or comorbidity, potentially masking subtle differences in outcomes. Reporting of DOAC plasma levels or reversal agent use was infrequent, precluding detailed subgroup analysis. The certainty of evidence ranged from very low to moderate across outcomes (Table 2), with most key estimates in the low-to-moderate range. Because outcome definitions for bleeding, transfusion thresholds, and operative delay windows were inconsistently reported and not directly harmonisable across studies, formal definition-restricted sensitivity analyses were not feasible; therefore, these outcomes were interpreted cautiously and downgraded accordingly in the GRADE assessment.

Implications for practice and research

Clinically, these findings support the safety of early surgery in DOAC-treated patients, provided renal function is adequate and no active bleeding is present. Routine surgical postponement based solely on DOAC use appears unwarranted. Multidisciplinary protocols that emphasise early fixation, individualised bleeding risk assessment, and standardised decision-making could reduce unnecessary delays and improve patient flow in trauma units. Future studies should focus on prospective, multicentre data collection incorporating DOAC-level assays, renal function stratification, and standard definitions for delay and bleeding severity. Pragmatic randomised trials comparing early versus delayed surgery under DOAC exposure remain feasible and would substantially strengthen the evidence base for guideline development.

## Conclusions

This systematic review and meta-analysis found that DOAC use in orthopaedic trauma is not associated with increased perioperative bleeding, transfusion, thromboembolic events, or mortality. However, DOAC exposure remains linked to moderate surgical delays, reflecting institutional caution rather than pharmacological necessity. These findings suggest that, in the absence of renal impairment or active bleeding, early operative fixation can generally proceed safely without routine pharmacological reversal. Adoption of standardised early-surgery pathways and clinician education on DOAC pharmacokinetics may help minimise unnecessary postponements, improve outcomes, and enhance efficiency within trauma care systems.

## Appendices

Electronic search strategy

Title/abstract and full-text screening were conducted in Rayyan [13].

**Table 3:** Electronic search strategy.

Database/Platform	Search strategy (verbatim as executed)	Limits/Filters	Last search date
MEDLINE (via PubMed)	Use the following blocks, then combine as: #1 AND #2 AND #3 #1 – Direct oral anticoagulants (DOACs) (“direct oral anticoagulant*”[tiab] OR DOAC*[tiab] OR NOAC*[tiab] OR “non-vitamin K antagonist oral anticoagulant*”[tiab] OR “novel oral anticoagulant*”[tiab] OR “target-specific oral anticoagulant*”[tiab] OR apixaban[tiab] OR rivaroxaban[tiab] OR dabigatran[tiab] OR edoxaban[tiab] OR betrixaban[tiab] OR ((“factor xa inhibitor*”[tiab]) AND oral[tiab]) OR ((“thrombin inhibitor*”[tiab]) AND oral[tiab]) OR “Apixaban”[Mesh] OR “Rivaroxaban”[Mesh] OR “Dabigatran”[Mesh] OR “Edoxaban”[Supplementary Concept] OR “Betrixaban”[Supplementary Concept] OR “Factor Xa Inhibitors”[Mesh] OR “Thrombin Inhibitors”[Mesh]) #2 – Orthopaedic trauma / fractures (orthop?edic*[tiab] OR trauma*[tiab] OR injur*[tiab] OR fracture*[tiab] OR “hip fracture*”[tiab] OR “neck of femur”[tiab] OR “femoral neck”[tiab] OR intertrochanteric[tiab] OR subtrochanteric[tiab] OR acetabular[tiab] OR pelvic[tiab] OR periprosthetic[tiab] OR “distal radius”[tiab] OR humer*[tiab] OR tibial*[tiab] OR ankle*[tiab] OR wrist*[tiab] OR “Fractures, Bone”[Mesh] OR “Hip Fractures”[Mesh] OR “Wounds and Injuries”[Mesh]) #3 – Perioperative / surgical (perioperative[tiab] OR preoperative[tiab] OR intraoperative[tiab] OR postoperative[tiab] OR surg*[tiab] OR operation*[tiab] OR operative[tiab] OR fixation[tiab] OR arthroplast*[tiab] OR “time to surgery”[tiab] OR delay*[tiab] OR “Perioperative Care”[Mesh] OR “Surgical Procedures, Operative”[Mesh] OR “Fracture Fixation”[Mesh] OR “Arthroplasty, Replacement, Hip”[Mesh]) Combine: #1 AND #2 AND #3	Publication years: 2010–2025 Language: English Species: Humans	01 October 2025
Embase (via Ovid)	1. (direct oral anticoagulant* or DOAC* or NOAC* or non-vitamin k antagonist oral anticoagulant* or novel oral anticoagulant* or target-specific oral anticoagulant*).ti,ab,kw. 2. (apixaban or rivaroxaban or dabigatran or edoxaban or betrixaban).ti,ab,kw. 3. ((factor xa adj3 inhibitor*) and oral).ti,ab,kw. 4. ((thrombin adj3 inhibitor*) and oral).ti,ab,kw. 5. exp apixaban/ or exp rivaroxaban/ or exp dabigatran/ or exp edoxaban/ or exp betrixaban/ 6. exp factor xa inhibitor/ or exp thrombin inhibitor/ 7. 1 or 2 or 3 or 4 or 5 or 6 8. (orthop?edic* or trauma* or injur* or fracture* or “hip fracture*” or “neck of femur” or “femoral neck” or intertrochanteric or subtrochanteric or acetabular or pelvic or periprosthetic or “distal radius” or humer* or tibial* or ankle* or wrist*).ti,ab,kw. 9. exp fracture/ or exp hip fracture/ or exp wound/ or exp multiple trauma/ 10. 8 or 9 11. (perioperat* or preoperat* or intraoperat* or postoperat* or surg* or operation* or operative or fixation or arthroplast* or “time to surgery” or delay*).ti,ab,kw. 12. exp perioperative period/ or exp surgery/ or exp fracture fixation/ or exp hip arthroplasty/ 13. 11 or 12 14. 7 and 10 and 13 15. limit 14 to yr= “2010 -Current” 16. limit 15 to english language 17. limit 16 to human	Publication years: 2010–2025 Language: English Species: Humans	01 October 2025
Cochrane CENTRAL (via Cochrane Library)	Use Advanced Search → Search Manager #1 – DOACs ("direct oral anticoagulant*“:ti,ab,kw OR DOAC*:ti,ab,kw OR NOAC*:ti,ab,kw OR “non-vitamin K antagonist oral anticoagulant*”:ti,ab,kw OR “novel oral anticoagulant*”:ti,ab,kw OR “target-specific oral anticoagulant*”:ti,ab,kw OR apixaban:ti,ab,kw OR rivaroxaban:ti,ab,kw OR dabigatran:ti,ab,kw OR edoxaban:ti,ab,kw OR betrixaban:ti,ab,kw) OR [mh “Factor Xa Inhibitors”] OR [mh “Thrombin Inhibitors”] OR [mh “Apixaban”] OR [mh “Rivaroxaban”] OR [mh “Dabigatran”] #2 – Orthopaedic trauma / fractures (orthop?edic*:ti,ab,kw OR trauma*:ti,ab,kw OR injur*:ti,ab,kw OR fracture*:ti,ab,kw OR “hip fracture*”:ti,ab,kw OR “neck of femur”:ti,ab,kw OR “femoral neck”:ti,ab,kw OR intertrochanteric:ti,ab,kw OR subtrochanteric:ti,ab,kw OR acetabular:ti,ab,kw OR pelvic:ti,ab,kw OR periprosthetic:ti,ab,kw OR “distal radius”:ti,ab,kw OR humer*:ti,ab,kw OR tibial*:ti,ab,kw OR ankle*:ti,ab,kw OR wrist*:ti,ab,kw) OR [mh “Fractures, Bone”] OR [mh “Hip Fractures”] OR [mh “Wounds and Injuries”] #3 – Perioperative / surgical (perioperative:ti,ab,kw OR preoperative:ti,ab,kw OR intraoperative:ti,ab,kw OR postoperative:ti,ab,kw OR surg*:ti,ab,kw OR operation*:ti,ab,kw OR operative:ti,ab,kw OR fixation:ti,ab,kw OR arthroplast*:ti,ab,kw OR “time to surgery”:ti,ab,kw OR delay*:ti,ab,kw) OR [mh “Perioperative Care”] OR [mh “Surgical Procedures, Operative”] OR [mh “Fracture Fixation”] OR [mh “Arthroplasty, Replacement, Hip”] Combine: #1 AND #2 AND #3		
